# Fatal Intracardiac Embolism Following Cement‐Augmented Pedicle Screw Instrumentation: A Case Report

**DOI:** 10.1155/cric/7883797

**Published:** 2026-09-12

**Authors:** Raul Eduardo Reyes Toledo, Laura Catalina Arcos, Jorge Alberto Carrillo

**Affiliations:** ^1^ Department of Cardiology, Division of Clinical Medicine, Hospital Universitario Méderi, Bogotá, Colombia; ^2^ Department of Radiology, Division of Diagnostic Imaging, Hospital Universitario Méderi, Bogotá, Colombia

## Abstract

**Background:**

Cement‐augmented pedicle screw instrumentation (CAPSI) is a widely used surgical technique for spinal stabilization and decompression, particularly in patients with poor bone quality. Although generally considered safe, cement extravasation can result in severe complications, including neurological injury and embolic events involving pulmonary or cardiac structures. Intracardiac cement embolism is a rare but potentially fatal occurrence.

**Case Presentation:**

A 67‐year‐old man with a history of hypertension and advanced renal cancer under palliative care presented with a 1‐week history of severe lumbar pain radiating to the right lower limb, accompanied by progressive weakness in both legs. Magnetic resonance imaging revealed an infiltrative soft tissue mass extending from T12 to L2 with spinal canal compression and metastatic‐appearing sacroiliac bone lesions. The patient underwent open posterior decompression with CAPSI using polymethylmethacrylate. Within 24 h postoperatively, he developed acute dyspnea and oppressive retrosternal chest pain. A contrast‐enhanced chest CT revealed hyperdense material consistent with cement within the inferior vena cava, pulmonary arteries, and right ventricle, with perforation of the posterior ventricular wall and extravasation into the anterior esophageal wall. Transesophageal echocardiography confirmed a 53‐mm linear, hyperechoic structure spanning the right atrium and perforating the anterior leaflet of the tricuspid valve, resulting in significant tricuspid regurgitation. A second cement fragment, located outside the atrial cavity, was associated with a mild pericardial effusion. Surgical intervention was recommended but declined by the patient. He subsequently developed obstructive shock due to cardiac tamponade and died.

**Discussion:**

This case highlights a rare but catastrophic complication of CAPSI. Accidental cement migration into the venous system can lead to cardiac perforation, even after an apparently uneventful surgical procedure. Prompt clinical suspicion and multimodal imaging are critical to identifying and managing this potentially fatal condition.

## 1. Introduction

Cement‐augmented pedicle screw instrumentation (CAPSI) is a surgical technique that has been used for over five decades, primarily in spinal stabilization and decompression procedures. The injection of bone cement of polymethylmethacrylate (PMMA) is employed to enhance pedicle screw fixation, particularly in patients with poor bone quality, and to reduce the risk of screw loosening or migration [1, 2]. However, the use of bone cement may lead to complications related to its extravasation, either into the intraspinal space or through the vertebral venous system, potentially resulting in neurological deficits or pulmonary and cardiac embolic events [3]. Unlike procedures such as vertebroplasty or kyphoplasty, cases of intracardiac embolism secondary to CAPSI are rarely reported in the current literature [4, 5].

We present a rare but clinically significant case of fatal cardiac embolism following CAPSI, emphasizing the need for early recognition and risk mitigation strategies in similar procedures

## 2. Case

A 67‐year‐old man with a history of essential hypertension and advanced‐stage renal cancer under palliative care presented with a three‐month history of severe lumbar pain radiating to the medial aspect of the right lower limb. His symptoms were associated with progressive weakness in both lower limbs, resulting in significant functional impairment and the need for a walking aid. He was evaluated by the neurosurgery team, and imaging studies revealed an extradural mass extending from T11 to L2, associated with a pathological vertebral fracture and severe spinal canal compression.

Given the clinical presentation, including progressive pain, paraparesis, and marked functional limitation, along with imaging findings suggestive of mechanical instability and significant neural compression, the patient was considered at high risk of further neurological deterioration. In this context, the neurosurgical team determined that surgical decompression and stabilization were indicated. Despite the patient′s advanced oncological condition, the procedure was performed with palliative intent, aiming at relieving pain, preserving neurological function, and improving quality of life.

In view of the high risk of further neurological deterioration, the patient underwent a minimally invasive spinal stabilization procedure with CAPSI. Cement augmentation was performed using PMMA bone cement (Mendec Spine Kit, Equipsalut). Under fluoroscopic guidance, percutaneous placement of cannulated transpedicular screws was achieved at T11, T12, L2, and L3. Following screw placement, PMMA cement was injected through the cannulated screws to enhance fixation in the setting of structurally compromised vertebral bone.

Although specific details regarding cement viscosity and total injected volume were not available in the operative report, cement augmentation was performed using a standard percutaneous technique under fluoroscopic guidance. The bone cement used is characterized by time‐dependent viscosity, transitioning from a low‐ to high‐viscosity state during polymerization.

**Figure 1:**
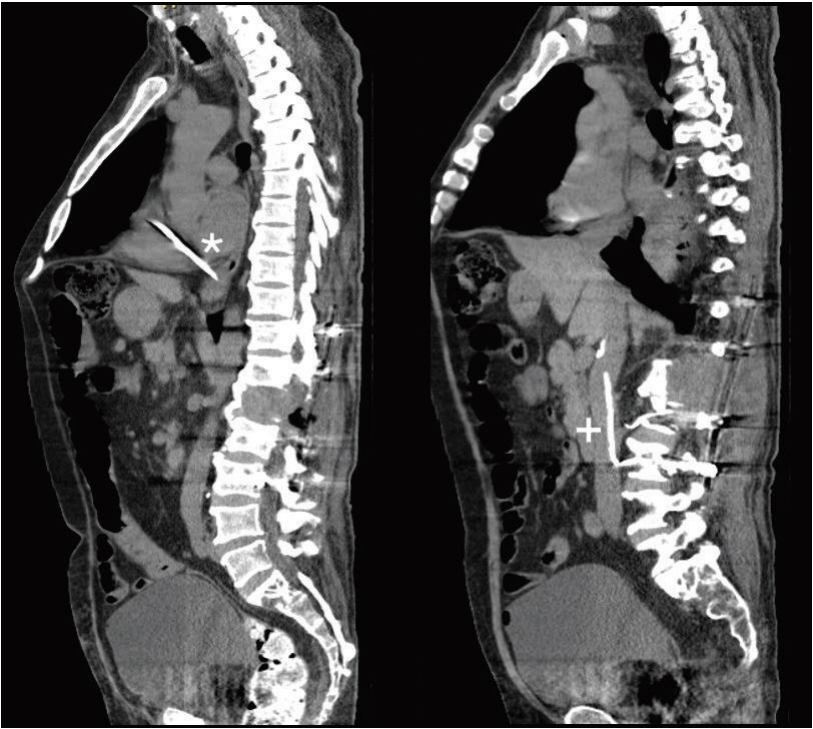
Sagittal computed tomography scan showing an infiltrative extradural lesion affecting the vertebral bodies from T11 to L2, consistent with metastatic spinal cord compression. Additionally, two hyperdense linear structures consistent with embolized cement are seen: one perforating the right ventricle (∗) and another ascending through the inferior vena cava from the lumbar venous plexus (+).

Within the first 24 h postoperatively, the patient developed progressive dyspnea, respiratory distress, oxygen desaturation, and retrosternal oppressive chest pain. A pulmonary embolism was suspected, prompting a contrast‐enhanced chest CT and admission to the intensive care unit. In the noncontrast phase of the study, hyperdense material was identified within both pulmonary arteries, consistent with cement embolization. A linear high‐density structure was also observed within the right atrium, extending through the posterior wall of the right ventricle and in close proximity to the anterior wall of the distal esophagus. Additionally, a longitudinal hyperdense structure measuring approximately 72 mm was detected in the infrarenal inferior vena cava, extending toward the paravertebral venous system and in contact with the L4 vertebral body (Figure 1).

**Figure 2:**
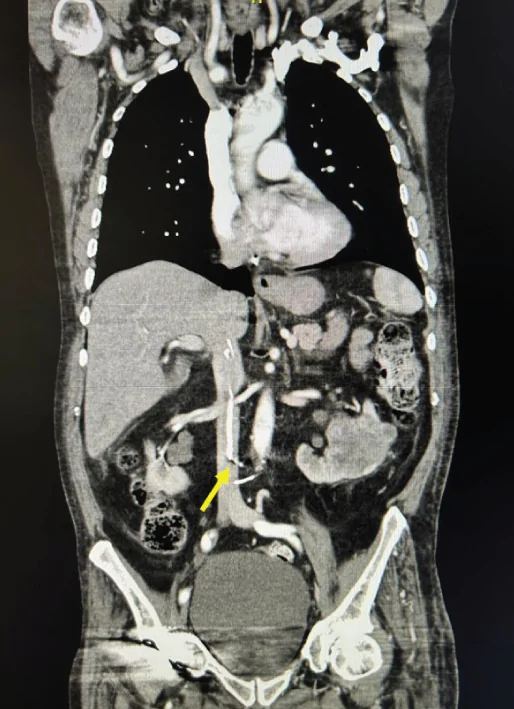
Coronal contrast‐enhanced computed tomography image showing a hyperdense linear structure within the inferior vena cava in continuity with the paravertebral venous plexus, consistent with intravascular cement migration (yellow arrow).

These findings demonstrated the presence of intravascular cement extending from the paravertebral región to the inferior vena cava, right heart chambers, and pulmonary arterial circulation, supporting a continuous distribution of embolized material along the venous system (Figure 2).

**Figure 3:**
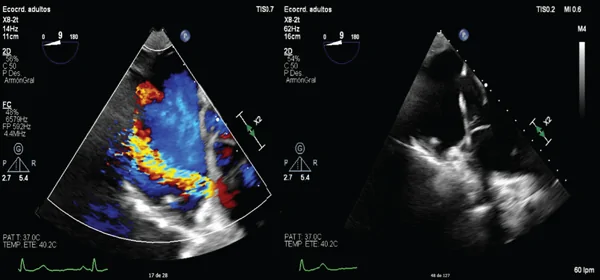
Mid‐esophageal transesophageal echocardiography view at 9° focused on the right heart chambers. A linear hyperrefringent structure is seen traversing the right atrium and perforating the anterior leaflet of the tricuspid valve, resulting in severe tricuspid regurgitation. Color Doppler imaging shows an eccentric regurgitant jet directed toward the lateral right atrial wall (Coanda effect).

**Figure 4:**
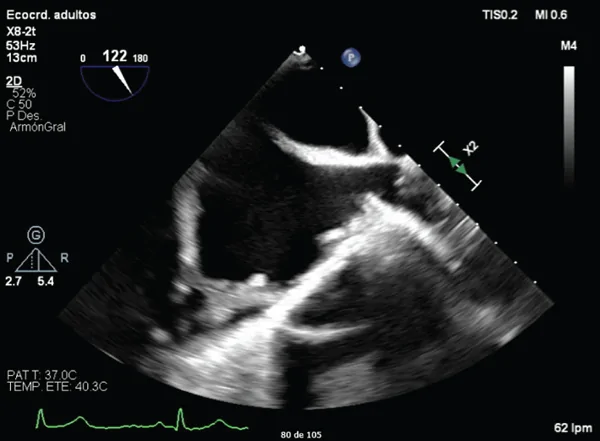
Bicaval transesophageal echocardiography view at 122°. A linear hyperrefringent structure is visualized outside the right atrial cavity, extending into the right ventricle and consistent with cement embolus. This finding is associated with a small pericardial effusion, without initial signs of cardiac tamponade.

A transesophageal echocardiogram was subsequently performed to better characterize the intracardiac embolism and confirm ventricular perforation. The echocardiographic assessment revealed a mildly dilated left ventricle with preserved systolic function (ejection fraction of 55%). A hyper‐reflective linear structure was observed within the right atrium, spanning the entire chamber, with an approximate length of 53 mm. Its distal end perforated the anterior leaflet of the tricuspid valve, producing two tricuspid regurgitation jets: one eccentric jet originating from the perforation site, with a Coanda effect along the right atrial wall, and a second central jet due to a coaptation defect. The proximal end of the structure extended toward the inferior atriocaval junction (Figure 3). A second fragment of material, measuring approximately 62 mm, was located outside the atrial cavity and was seen perforating the right ventricle, associated with a mild pericardial effusion without evidence of compressive physiology at the time of the study (Figure 4).

Given these findings, the cardiovascular surgery team proposed surgical intervention, including tricuspid valve replacement and extraction of the cement fragments. However, after being informed of the risks, the patient chose not to undergo surgery and signed a consent form refusing the procedure. He remained under close monitoring in the intensive care unit, receiving supportive management including oxygen therapy and opioid‐based analgesia. Anticoagulation was not initiated due to the presence of cardiac perforation and the associated risk of bleeding complications. Given the clinical context and the patient′s decision, a palliative approach focused on comfort measures was adopted, allowing family presence to support the patient during the end‐of‐life phase. A few hours later, he experienced rapid clinical deterioration due to cardiac tamponade and subsequently died.

## 3. Discussion

CAPSI is an effective technique frequently used to optimize spinal fixation in patients with poor bone quality, such as those with osteoporosis or neoplastic infiltrative lesions [6, 7]. The use of bone cement, typically PMMA, provides greater screw stability but carries potential complications related to the risk of extravasation into the spinal canal or vascular structures.

Globally, cement extravasation is recognized as a common complication of CAPSI procedures, with reports describing incidences ranging from 10% to 30% of cases [8, 9]. Although the exact mechanism of this extravasation is not fully understood, it has been postulated that the risk is primarily associated with bone‐related structural factors—such as porosity, permeability, and the size of the injected cavity—as well as with characteristics of the cement material itself, particularly its viscosity. The latter is considered one of the few potentially modifiable factors, with high‐viscosity cements being associated with a lower risk of leakage [10]. Additionally, several patient‐related risk factors have been identified, including the number of vertebral levels treated, the presence of bone metastases, preexisting cardiopulmonary disease, and a total cement volume exceeding 30 mL [11].

Cement extravasation can present with a wide range of clinical manifestations, from incidental findings on imaging studies to neurological symptoms secondary to neural structure involvement or embolic events in various locations—predominantly pulmonary, and less frequently, cardiac—with estimated incidences of 7.9% and 3.9%, respectively [12]. Only a small proportion of cases follow a symptomatic course, but when they do, they may be associated with severe complications and a high risk of mortality [13]. Cardiac cement embolism events are related to leakage that reaches the vertebral venous system and subsequently migrates to the inferior vena cava or azygos vein, ultimately reaching the right heart chambers. This process is facilitated by the vertebral venous plexus (Batson′s plexus), a valveless network that allows bidirectional blood flow and direct communication between the spinal venous system and the inferior vena cava, thereby enabling the migration of cement into the cardiopulmonary circulation [14].

In the present case, the CT findings (Figures 1 and 2) demonstrate a continuous linear cement column extending from the paravertebral venous plexus to the inferior vena cava, providing important clues to the underlying mechanism of embolization. This imaging pattern suggests that PMMA leaked into the vertebral venous system while still in a low‐viscosity state. The cement likely underwent in situ polymerization within the venous lumen, forming a rigid intravascular cast that subsequently detached and embolized to the right heart [15]. Although inadvertent direct venous injection cannot be completely excluded, the imaging appearance in this patient is more consistent with progressive venous leakage followed by in situ polymerization and subsequent embolization.

Life‐threatening complications include acute respiratory distress syndrome, pericardial effusion, or cardiac perforation, often preceded by the sudden onset of dyspnea, oxygen desaturation, hypotension, tachycardia, or chest pain [16]. Symptoms can occur at any time during the postoperative period, typically within the first few days after surgery. However, cases have been documented with delayed presentation, even up to 5 years postintervention [17]. In the present case, symptoms had an acute onset, with rapid clinical deterioration due to acute right heart failure caused by traumatic perforation of the anterior leaflet of the tricuspid valve and the right ventricle, with secondary cardiac tamponade that ultimately led to death.

The management of cardiac cement embolism primarily depends on the presence of symptoms and associated complications. In asymptomatic patients, a conservative approach is usually adopted. In contrast, cases involving cardiac perforation with hemodynamic compromise clearly require surgical treatment, aiming to repair the damaged tissues and remove the embolized material [18].

Although anticoagulation has been proposed in selected cases of pulmonary cement embolism to prevent secondary thrombus formation over the foreign material, this strategy is based on the potential for endothelial injury and activation of the coagulation cascade induced by the intravascular presence of PMMA. However, its use remains individualized and is supported by limited evidence, with no standardized recommendations available [19].

In cases of intracardiac embolism with associated mechanical complications, such as valvular injury or cardiac perforation, as observed in this case, anticoagulation is generally not favored, as the underlying mechanism is primarily structural rather than thrombotic and the risk of bleeding—particularly in the presence of pericardial effusion or myocardial injury—may outweigh any theoretical benefit [13].

In this instance, the patient declined surgical intervention after being informed of the risks. His decision was respected, and a palliative care plan was implemented, focused on pain control and comfort measures.

## 4. Conclusion

This case is aimed at highlighting the importance of maintaining a high level of clinical suspicion for cardiopulmonary cement embolism in patients who present with clinical deterioration during the postoperative period following CAPSI procedures. Additionally, it underscores the need to identify both patient‐related risk factors and technical aspects of the procedure in order to mitigate the development of this potentially fatal complication.

## Funding

No funding was received for this manuscript.

## Consent

Written informed consent was not obtained as the case report does not contain any identifiable personal information and complies with the ICMJE guidelines for anonymized case reporting. The case was reviewed and approved for publication by the Research Department of Mederi Hospital in Bogotá, Colombia.

## Conflicts of Interest

The authors declare no conflicts of interest.

## Supporting information


**Supporting Information** Additional supporting information can be found online in the Supporting Information section. Video S1: Transesophageal echocardiography (mid‐esophageal right ventricular inflow–outflow view) demonstrating a hyperechogenic linear structure within the right ventricle consistent with intracardiac cement embolism, with associated significant tricuspid regurgitation on color Doppler.

## Data Availability

Data sharing is not applicable to this article as no datasets were generated or analyzed during the current study.
